# *BRAF* Exon 15 Mutations in Papillary Carcinoma and Adjacent Thyroid Parenchyma: A Search for the Early Molecular Events Associated with Tumor Development

**DOI:** 10.3390/cancers12020430

**Published:** 2020-02-12

**Authors:** Giorgia Acquaviva, Dario de Biase, Chiara Diquigiovanni, Chiara Maria Argento, Antonio De Leo, Elena Bonora, Kerry Jane Rhoden, Annalisa Pession, Giovanni Tallini

**Affiliations:** 1 Department of Experimental, Diagnostic and Specialty Medicine, University of Bologna-Molecular Diagnostic Unit, Azienda USL di Bologna, 40138 Bologna, Italy; giorgia.acquaviva3@unibo.it (G.A.); antonio.deleo@unibo.it (A.D.L.); 2 Department of Pharmacy and Biotechnology, University of Bologna-Molecular Diagnostic Unit, Azienda USL di Bologna, 40138 Bologna, Italy; dario.debiase@unibo.it (D.d.B.); chiaramaria.argento@studio.unibo (C.M.A.); annalisa.pession@unibo.it (A.P.); 3 Genetics Unit, Department of Medical and Surgical Sciences, University of Bologna, S. Orsola-Malpighi Hospital, 40138 Bologna, Italy; chiara.diquigiovanni@unibo.it (C.D.); elena.bonora6@unibo.it (E.B.); kerry.rhoden@unibo.it (K.J.R.)

**Keywords:** papillary thyroid carcinoma, *BRAF* exon 15 mutations, BRAF p.V600E, tumor development, follicular cell atypia, follicular cell hyperplasia, psammoma bodies, massively parallel sequencing

## Abstract

*BRAF* exon 15 mutations are the most common molecular alterations found in papillary thyroid carcinoma (PTC). To date, there is no information regarding *BRAF* alterations in the thyroid parenchyma surrounding the tumor. To explore the early events associated with the development of PTC, we used massively parallel sequencing to investigate *BRAF* exon 15 in 30 PTCs and in 100 samples from the thyroid parenchyma surrounding the tumor. BRAF p.V600E was identified in 19/30 PTCs (63.3%). BRAF p.V600E mutations were identified in the tissue adjacent the PTC only in samples containing psammoma bodies. The other samples were either *BRAF* wild type (WT) or carried BRAF non p.V600E mutations. Specifically, BRAF p.G593D, -p.A598T, -p.V600M, -p.R603Q, -p.S607F, and -p.S607P were identified in 4 of 36 (11.1%) samples with follicular cell atypia, in 2 of 16 (12.5%) with follicular cell hyperplasia, and in 1 of 33 (3.0%) histologically normal samples—Only in tissue surrounding BRAF p.V600E mutated PTCs. These mutations are predicted to affect protein function in silico but, in vitro, have kinase activity and BRAF phosphorylation levels similar to BRAF WT. No *BRAF* exon 15 mutations were identified in samples adjacent to PTCs that were *BRAF* WT. A mutagenic process affecting *BRAF* exon 15 occurs in a subset of thyroid glands that develop BRAF p.V600E mutated PTCs.

## 1. Introduction

Papillary thyroid carcinoma (PTC) is the most common malignancy of the endocrine system [1], and its incidence has been increasing in the recent past, mostly due to the early detection of small asymptomatic tumors [1,2,3]. The molecular signature of PTC has been extensively investigated, and a remarkable correlation between molecular alterations and PTC histologic phenotypes (i.e., PTC histotype variants) has become apparent [2,4]. The work of the Cancer Genome Atlas classification of PTC [4] has identified two molecular signatures: (i) *BRAF p.V600E-like* typical of classic/conventional PTC and (ii) *RAS-like* typical of the encapsulated follicular variant PTC, a tumor type with features that overlaps with other follicular patterned tumors, such as follicular thyroid adenoma and carcinoma [2,4,5]. *BRAF* exon 15 mutations are present in 40% to 80% of PTC, and the vast majority of them—i.e., greater than 95%—consist in a point mutation that involves nucleotide 1799 (thymine to adenine trans-version, c.1799T>A). The resulting valine to glutamate substitution at residue 600 (BRAF p.V600E) mimics BRAF phosphorylation, causing constitutive activation of the protein [2]. Other far less common *BRAF* alterations include nucleotide changes associated with follicular patterned tumors (follicular adenoma, follicular carcinoma, and encapsulated follicular variant papillary carcinoma) and rearrangements associated with radiation-induced PTCs [2].

BRAF p.V600E mutation, like other single-nucleotide changes in *BRAF* or other genes relevant for thyroid tumorigenesis (e.g., *H-*, *K-*, and *N-RAS*), are supposed to result from exposure to chemical carcinogens, although very little is known about the process that leads to the occurrence of these simple genetic alterations [2]. In spite of the very high specificity of BRAF p.V600E for PTC [2], the *BRAF* exon 15 status of thyroid tissue surrounding the tumor is unknown. 

To test the mutagenic process that leads to BRAF p.V600E mutated PTCs, we used massively parallel sequencing to evaluate the *BRAF* exon 15 status in 100 thyroid tissue samples adjacent to 30 PTCs, with the aim of better understanding whether potential precursor lesions are associated with the development of PTC.

## 2. Results

### 2.1. Clinicopathologic Features of Tumors and Histologic Features of Adjacent Thyroid Tissue

**Figure 1:**
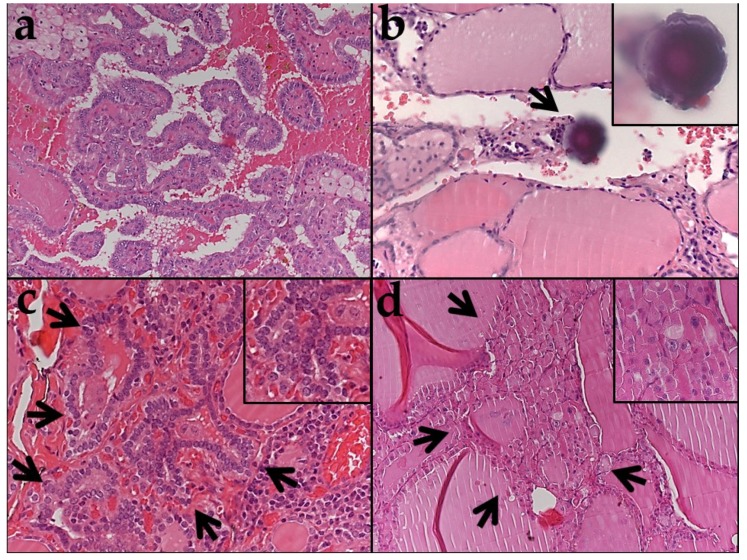
Histologic appearance of the samples analyzed (**a**–**d**). (**a**) Papillary carcinoma BRAF p.V600E mutated (×100). (**b**) Psammoma body (arrow) protruding into a dilated lymphatic space (×100); the inset shows the psammoma body at higher magnification and in the plane of focus (×400). BRAF p.V600E is identified after sequencing of DNA extracted from the area containing the psammoma body. (**c**) Focus of follicular cell atypia (×200) (arrows, inset at higher magnification, ×400). BRAF p.V600M is identified after the sequencing of DNA extracted from the area with follicular cell atypia. (**d**) Focus of follicular cell hyperplasia with oncocytic change (×200) (arrows, inset at higher magnification, ×400). BRAF p. S607P is identified after the sequencing of DNA extracted from the area with follicular cell hyperplasia.

**Figure 2:**
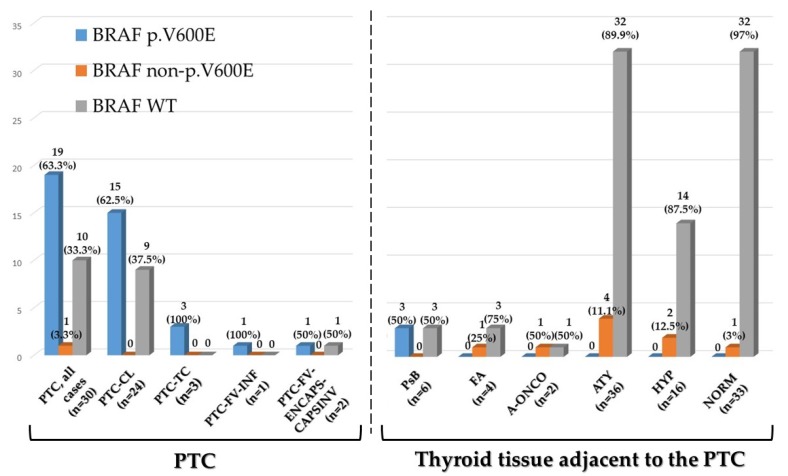
*BRAF* exon 15 status in thyroid samples. Bars represent the number of samples that are BRAF p.V600E, BRAF non p.V600E mutated, and *BRAF* wild type (WT). Percentages are in parenthesis. Two separate samples were analyzed from one follicular adenoma for a total of four samples from three follicular adenomas. Two separate samples were analyzed from one oncocytic follicular adenoma. PTC: papillary thyroid carcinoma; PTC-CL: classic PTC; PTC-TC: PTC, tall cell variant; PTC-FV-INF: PTC follicular variant—Infiltrative; PTC-FV-ENCAPS-CAPSINV: PTC follicular variant—Encapsulated, with capsular invasion; PsB: psammoma bodies; FA: follicular adenoma; FA-ONC: follicular adenoma, oncocytic; ATY: thyroid tissue with follicular cell atypia; HYP: thyroid tissue with follicular cell hyperplasia; and NORM: histologically normal thyroid tissue.

Table S1 is a list of all samples analyzed. The clinicopathologic characteristics of the 30 PTCs are summarized in Table 1. Most tumors were classic papillary carcinomas (Figure 1a). One case was classified as infiltrative follicular variant PTC, a type of PTC that is known to have a *BRAF p.V600E-like* profile [2]. Two cases were classified as encapsulated follicular variant papillary carcinoma, a type of PTC known to have an *RAS-like* molecular profile [2,4,6,7], and both had invasion of the tumor capsule. Thyroid tissue from the parenchyma surrounding the PTC was selected from areas that were histologically normal (34 samples), that had follicular cell atypia (38 samples) or follicular cell hyperplasia (16 samples), that contained psammoma bodies in the absence of tumor cells as seen under the microscope (6 samples), or from follicular adenomas (one of which was oncocytic) that coexisted with a PTC in four of the thirty thyroidectomy specimens (Table 1 and Figure 2; we analyzed two samples from the oncocytic follicular adenoma, two samples from one follicular adenoma, and one sample each from the remaining two follicular adenomas). Psammoma bodies are round calcific tridimensional structures with distinctive concentric laminations found in the thyroid stroma or in the vicinity of lymphatic vessels (Figure 1b) that are considered a histologic marker for PTC [6]. The areas of follicular cell atypia (Figure 1c) were defined by the presence of unusual features, including follicular cell crowding and minor alterations of the nuclear morphology, such as mild nuclear clearing, mild enlargement, and contour irregularities, similar to what is described in multifocal fibrosing (sclerosing) thyroiditis [7]. The areas of follicular cell hyperplasia (Figure 1d) consisted of millimetric foci of increased cellular density with microfollicles and enlarged follicular cells similar to those observed in the hyperplastic nodules of multinodular goiter [6].

### 2.2. BRAF Exon 15 Mutations in Papillary Carcinoma

The results are summarized in Table S1, Table 2, and Figure 2. One representative case with a BRAF p.V600E mutation is illustrated in Figure 1a. Overall, the BRAF p.V600E mutation was identified in 19/30 cases (63.3%), including 15/24 classic PTCs (62.5%), all tall cell variant PTCs (3/3, 100%), and the only infiltrative follicular variant PTC (1/1, 100%). The only PTC with a BRAF non p.V600E mutation (Case 21, Table S1) was BRAF p.V600A mutated. BRAF p.V600A is a *RAS-like BRAF* exon 15 mutation according to the Cancer Genome Atlas classification of papillary thyroid carcinoma [4] and is compatible with the diagnosis of encapsulated follicular variant papillary carcinoma.

### 2.3. BRAF Exon 15 Mutations in Thyroid Tissue Adjacent to Papillary Carcinoma

**Figure 3:**
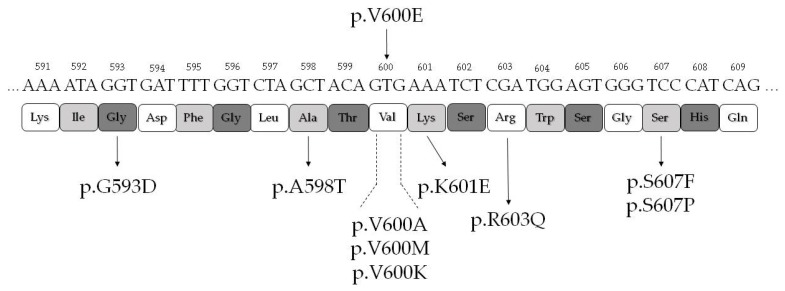
Schematic drawing of *BRAF* exon 15 mutations. The position and residue substitutions of all mutations identified are shown with respect to the standard *BRAF* nucleotide, codon, and amino acid residue sequence.

One hundred samples from thyroid tissue surrounding the PTCs were analyzed. The results are summarized in Table S1, Table 2, Table 3, Table 4, Figure 2, and Figure 3; representative cases are illustrated in Figure 1b–d. In these cases, BRAF p.V600E was identified in three of six samples containing psammoma bodies (without viable tumor cells) (Table 2, Figure 1b, and Figure 2); in all three cases, BRAF p.V600E was also present in the PTC (Table S1). The three samples with psammoma bodies without *BRAF* mutations were obtained from thyroid tissue surrounding PTCs that were *BRAF* WT (Table S1). 

Thus, there was a perfect correspondence between *BRAF* exon 15 status of the PTC and of the psammoma bodies surrounding the tumor. The other samples from thyroid tissue adjacent to the PTC were either *BRAF* exon 15 WT or carried BRAF non p.V600E mutations. The overall correlation between BRAF p.V600E and psammoma bodies in the samples analyzed was statistically significant (*p* = 0.012; chi-square test).

All the BRAF non p.V600E mutations were identified between residues 593 and 607 (Figure 3). BRAF non p.V600E mutations were identified in two of four follicular adenomas that coexisted with the PTC (Table 2 and Figure 2). One follicular adenoma was BRAF p.K601E mutated, while the corresponding PTC was *BRAF* WT (Case 28 of Table S1, Table 3, and Figure 2). One oncocytic follicular adenoma carried two subclonal *BRAF* exon 15 mutations: p.V600K and p.S607P (Case 4 of Table S1, Table 3, and Figure 2), while the corresponding PTC was BRAF p.V600E mutated. Interestingly, BRAF p.S607P was also identified in a focus of follicular cell hyperplasia with oncocytic change adjacent to the oncocytic adenoma (Case 4 of Table S1 and Figure 1d). However, the majority of BRAF non p.V600E mutations were identified not in the adenomas—coexisting with the PTC as an incidental finding in the thyroidectomy specimen—but in the non neoplastic tissue adjacent to the PTC (Table 2, Table 3, and Figure 2). BRAF non p.V600E were present in 4 of 36 (11.1%) samples with follicular cell atypia, in 2 of 16 (12.5%) samples with follicular cell hyperplasia, and in 1 of 33 (3.0%) histologically normal samples. Importantly, if we exclude the cases of adenomas coexisting with the PTC, all BRAF non p.V600E mutations were identified only in tissue adjacent to PTCs harboring the BRAF p.V600E mutation. No *BRAF* exon 15 mutation was identified in thyroid samples surrounding PTCs that were *BRAF* wild type (with the exception of a BRAF p.K601E mutation present in a follicular adenoma coexisting with the PTC) (Case 28 of Table S1 and Table 4). We thus postulated that the restriction of BRAF non p.V600E mutations in thyroid tissue adjacent to PTCs p.V600E mutated may have biological significance. In order to understand whether the BRAF non p.V600E mutations we identified affect BRAF activity, we used PolyPhen-2 (Polymorphism Phenotyping v2) to assess the effect of the mutations in silico. All mutations were predicted as damaging, with the exception of BRAF p.S607P (Table 3).

### 2.4. Functional Characterization of MAPK Signaling by BRAF Non p.V600E Mutant Proteins

**Figure 4:**
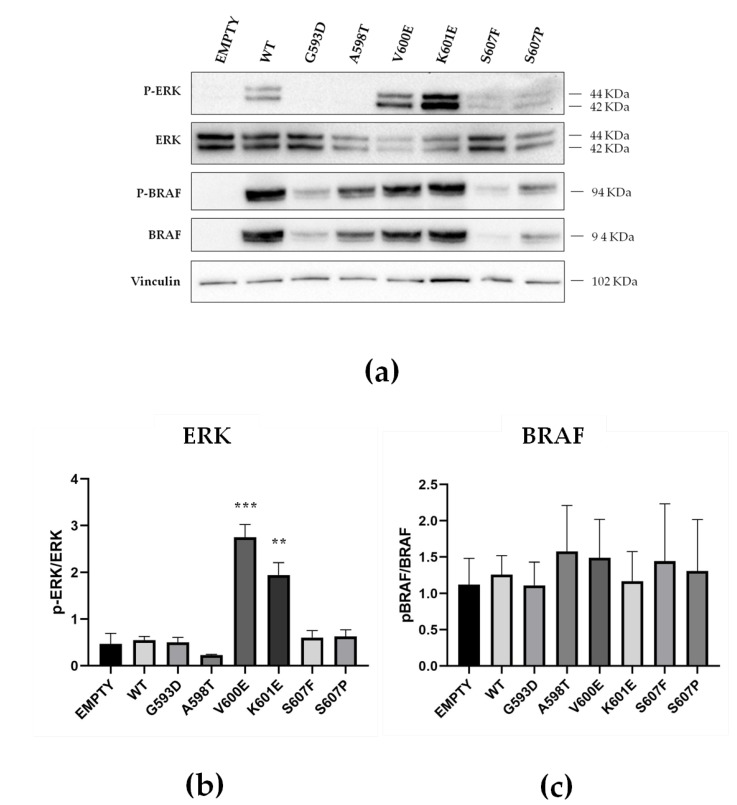
Functional characterization of BRAF p.G593D, BRAF p.A598T, BRAF p.S607F, and BRAF p.S607P compared to BRAF wild type (WT), BRAF p.V600E, BRAF p.K601E, and empty vector (EMPTY). (**a**) Effect of BRAF mutation on the MAPK signaling pathway: representative Western Blot of ERK, p-ERK, BRAF, and p-BRAF proteins in HEK-293 cells transiently transfected with wild type or mutant BRAF plasmids. Vinculin was used as a loading control. (**b**) Effect of BRAF mutation on ERK1/2 phosphorylation: densitometric analysis of Western Blots showing the p-ERK/ERK ratio in HEK-293 cells transiently transfected with wild type and mutant BRAF plasmids. HEK-293 cells transfected with BRAF-V600E and BRAF-K601E show increased activation of the MAPK pathway compared to BRAF WT, whereas HEK-293 cells transfected with BRAF-G593D, BRAF-A598T, BRAF-S607F, and BRAF-S607P show similar MAPK activation to BRAF WT. Data have been normalized to nontransfected controls (not shown); data are reported as the mean ± SEM of at least two independent experiments. **: *p* < 0.005 and ***: *p* < 0.0001. (**c**) Effect of BRAF mutation on BRAF phosphorylation: densitometric analysis of Western Blots showing the p-BRAF/BRAF ratio in HEK-293 cells transiently transfected with wild type and mutant BRAF plasmids. There are no significant differences in BRAF phosphorylation levels between the different constructs. Data have been normalized to nontransfected control; data are reported as the mean ± SEM of at least two independent experiments.

To assess the functional activity of BRAF mutant proteins, ERK1/2 phosphorylation was evaluated in transiently transfected HEK-293 cells as an index of MAPK activation. In the MAPK signaling pathway, BRAF phosphorylates MEK1/2 at serine 217 and 221, and MEK1/2, in turn, phosphorylate ERK1/2 at threonine 202 and tyrosine 204 [4]. ERK1/2 phosphorylation in cells expressing BRAF non p.V600E mutated species found in the tissue adjacent to the PTC was compared to that of BRAF WT and BRAF p.V600E. ERK1/2 phosphorylation was higher in HEK-293 cells expressing BRAF p.V600E (5.6 fold, *p* < 0.0001) or BRAF p.K601E (3.9 fold, *p* < 0.005) when compared to cells expressing BRAF WT (Figure 4). In contrast, ERK1/2 phosphorylation by BRAF p.G593D, BRAF p.A598T, BRAF p.S607F, and BRAF p.S607P was similar to BRAF WT (Table S2, Figure 4, and **Figure S1**). Using the same HEK-293 in vitro model, we also analyzed BRAF phosphorylation in order to understand whether the BRAF non p.V600E mutations affect BRAF protein affinity for pyrophosphate. Interestingly, we observed that BRAF p.G593D, BRAF p.A598T, BRAF p.V600E, BRAF p.K601E, BRAF p.S607F, and BRAF p.S607P all showed BRAF phosphorylation levels similar to BRAF WT (Table S2, Figure 4, and Figure S1).

## 3. Discussion

In this study, we analyzed the mutational status of BRAF exon 15 in 100 samples from thyroid tissue surrounding 30 randomly selected PTCs to better understand the mutagenic process that leads to BRAF p.V600E mutated PTCs.

The prevalence and type of BRAF exon 15 mutations in our PTCs are compatible with those reported in the literature; BRAF p.V600E was identified in 62.5% of classic PTC, in all tall cell PTC variants, and in the only infiltrative follicular variant PTC, all tumor types associated with a *BRAF p.V600E-like* mutational profile [2]. The only PTC with a BRAF non p.V600E exon 15 mutation carried BRAF p.V600A, an *RAS-like BRAF* exon 15 mutation compatible with the diagnosis of encapsulated follicular variant papillary carcinoma [2]. 

Among the samples surrounding the PTCs, the only ones with BRAF p.V600E were those containing psammoma bodies—in the absence of identifiable tumor cells. In these samples, the presence of the mutation showed a perfect correspondence with that present in the PTC to which they were associated. The overall correlation between BRAF p.V600E and psammoma bodies in all the samples analyzed was statistically significant. Psammoma bodies have long been known to be a marker of PTC [8]. They are believed to develop from small groups or even single papillary carcinoma cells that, by undergoing necrosis, form a nidus for the deposition of successive layers of lamellated calcium salt deposits and are considered “the ghosts of dead papillae” [6,9]. The identification of BRAF p.V600E only in psammoma body samples surrounding BRAF p.V600E mutated papillary carcinoma is coherent with the hypothesis that the psammoma bodies in PTC originate indeed from dead papillary carcinoma cells.

The mutations found in the adenomas that coexisted with the PTCs in the same thyroid gland were *RAS-like BRAF* mutations [4], typical of follicular-patterned lesions, and—not surprisingly—were different from those of the PTCs to which they were associated. Specifically, the BRAF p.K601E mutation has been reported in follicular adenoma [10], while BRAF p.V600K has been reported in encapsulated follicular variant PTC [11]. Both mutations confer a gain of function to the BRAF protein, resulting in increased kinase activity [12].

Most BRAF non p.V600E exon 15 mutations were identified in the non neoplastic tissue adjacent to the PTC, and remarkably—after exclusion of the adenomas—all BRAF non p.V600E mutations were identified only in tissue adjacent to PTCs harboring the BRAF p.V600E mutation. They were present in approximately 10% of samples with follicular cell atypia histologically similar to that observed in multifocal fibrosing (sclerosing) thyroiditis [7] and in a similar proportion of samples with follicular cell hyperplasia. In a single case, a BRAF non p.V600E exon 15 mutation (p.S607F) was identified in histologically normal thyroid tissue. 

All BRAF non p.V600E exon 15 mutations identified belong to the enzymatic kinase domain of BRAF (Conserved Region 3, CR3) in the C-lobe (residues 535–717) that binds substrate proteins, with most mutations clustering in and around the DFG motif (D594, F595, and G596) and the activation loop (Residues 596–600) [13]. All of those found in non neoplastic samples have been previously reported in the literature and described in different types of tissues, including the thyroid gland. BRAF p.G593D has been identified in solid cell nest hyperplasia in association with BRAF p.V600E mutated PTC [14], while the BRAF p.R603Q and p.S607F mutations were reported in nevi, in association with BRAF p.V600E mutated melanomas [15], similar to what we observed in our cases. BRAF p.S607P and p.V600M have been reported in melanoma [12,16], while BRAF p.A598T has been identified in non small cell lung cancer [17].

MAPK signaling of BRAF p.G593D, BRAF p.A598T, BRAF p.S607F, and BRAF p.S607P found in non neoplastic samples was assessed in vitro. Kinase activity was compared to that of BRAF WT and to mutations well-known to lead to constitutive activation of the BRAF protein: BRAF p.V600E—with the highest kinase activity—and BRAF p.K601E [12]. Although our BRAF-mutated species were predicted to affect protein function in silico, all of them had kinase activity comparable to that of the BRAF WT protein, nor were they associated with increased phosphorylation levels of the mutated BRAF protein. It is tempting to speculate that, although our BRAF non p.V600E exon 15 mutations do not lead to increased MAPK pathway activation, they may induce the production of protumorigenic cytokines and chemokines in cells carrying the mutation. Indeed, a connection between cancer development and chemokines has been proposed in several tumors—including those of the thyroid gland [18,19,20,21,22,23,24,25,26]. Some cytokines like CXCL8 (also known as interleukin-8 or neutrophil attractant/activation protein-1) are emerging as important players in the modulation of the thyroid cancer microenvironment, with a role for the initiation and maintenance of tumorigenesis [21]. 

Taken together, our data indicate that a mutagenic process affecting *BRAF* exon 15 takes place in a subset of thyroid glands that develop BRAF p.V600E mutated PTCs. While the carcinoma develops only if the p.V600E mutation occurs, its development is accompanied by other exon 15 mutations. Our data suggest that p.V600E is one of a cluster of *BRAF* mutations occurring in the thyroid tissue of some patients. Only mutations with definite oncogenic potential like BRAF p.V600E lead to the development of PTC; others without oncogenic potential are present in adjacent non malignant tissues, where they produce minor histologic abnormalities such as follicular cell atypia or hyperplasia. The cause of this putative mutagenic process is unlikely related to modification of the inactivation of the DNA mismatch repair system that is altered only in a minority of poorly and undifferentiated thyroid carcinomas [2,18]. It may, however, be linked to exposure of the thyroid parenchyma to chemical carcinogens, as in the case of simple nucleotide changes in other genes—such as *H-*, *K-*, *N-RAS*—that are relevant for thyroid tumorigenesis [2,27]. The nature of this mutagenic process and its relevance for PTC development deserves further investigation.

## 4. Materials and Methods

### 4.1. Case Selection

Thirty PTCs were randomly selected from the archives of the Anatomic Pathology Units of the Bellaria and Maggiore Hospital in Bologna (Italy). Tumor samples and surrounding thyroid tissues were obtained from formalin-fixed and paraffin-embedded (FFPE) tissue blocks processed following routine diagnostic procedures. PTCs (including tumors measuring 1 centimeter or less, also known as papillary microcarcinomas) were subtyped according to current classification schemes [5]. The study was approved by the Iinternal Review Board of the University of Bologna Medical Center (number: 51/2017/O/OssN). Information regarding the human material was managed using anonymous codes, and all samples were handled in compliance with the Helsinki Declaration. Follow-up information was not used in this study. 

### 4.2. Detection of BRAF Mutations by Next Generation Sequencing

After routine hematoxylin and eosin (H&E) sections were cut for case review, two to four additional 10 μm thick sections were cut from each selected block, followed by one H&E control slide. To assure a strict histologic control for all samples analyzed, the area of interest was marked on this H&E control slide. The material for sequence analysis was then manually dissected under microscopic guidance from corresponding 10 μm sections using a sterile blade. All psammoma body samples used for molecular analysis were devoid of viable tumor cells.

DNA for mutation analysis was extracted using the HighPure PCR Template Preparation Kit (Roche-Diagnostic, Manheim, Germany) following protocols specified by the manufacturer. DNA concentration was measured using the Quant-iT™ dsDNA BR Assay Kit on the Qubit™ Quantitation Platform (ThermoFisher Scientific, Waltham, MA, USA). *BRAF* exon 15 was analyzed by next-generation sequencing (NGS) using the 454 GS-Junior (Roche Diagnostic) platform following the manufacturer’s instructions and according to previously reported protocols [28]. Briefly, at least 10 ng of DNA was amplified targeting *BRAF* exon 15 using the following primers: sense 5′-TGCTTGCTCTGATAGGAAAATGA-3′ and antisense 5′-TGGATCCAGACAACTGTTCAAA-3′ (Table 5). Primers were modified with universal tail sequences and multiple identifier (MID) nucleotides. After purification and quantification, emulsion-PCR and sequencing were performed according to the manufacturer’s protocol. Results were analyzed using Amplicon Variant Analyzer (AVA) software (Version 3.0, Roche Diagnostic, Mannheim, Germany). Only nucleotide variations observed in both strands with at least 5% of allele-read were considered for mutational calling. The presence of all BRAF non p.V600E mutations was confirmed by repeating twice the analysis with the Next Generation Sequencing platform.

### 4.3. Mutant BRAF Plasmids Generation via Site-Directed Mutagenesis

The pCMV6 construct encoding wild type *BRAF* (#40775) in frame with the FLAG tag was purchased from Addgene (Cambridge, MA, USA). Mutations—p.G593D, p.A598T, p.V600E, p.K601E, p.S607F, and p.S607P—were inserted using the Q5 Site direct Mutagenesis kit (New England Biolabs, Ipswich, MA, USA) according to manufacturer’s instructions. The oligonucleotides used for mutagenesis are reported in Table 5. Site-directed mutagenesis was verified by Sanger sequencing of plasmids using *BRAF* exon 15 targeting primers (see Table 5).

### 4.4. Cell Lines and Transfection

Cell models used were HEK-293 cells, and HEK-293 cells overexpressing wild type or mutant BRAF (p.G593D, p.A598T, p.V600E, p.K601E, p.S607F, and p.S607P). Cells were cultured in Dulbecco’s modified Eagle’s medium (DMEM, Euroclone, Milan, Italy) supplemented with 10% (v/v) fetal bovine serum, 2 mM L-glutamine, 100 U/mL penicillin, and 100 μg/mL streptomycin (Sigma-Aldrich, St. Louis, MO, USA) and grown in a humidified incubator at 37 °C with 5% CO_2_ in the air.

For transfection, 3.5 × 10^5^ cells HEK-293 were seeded in complete medium. Twenty-four hours later, 4 μg of *BRAF* mutant plasmids were transfected using liposomes according to manufacturer’s instructions (Lipofectamine 3000, Thermo Fisher Scientific, Grand Island, NY, USA). 

### 4.5. Western Blots

Forty-eight hours after transfection, HEK-293 cells were lysed in ice cold RIPA buffer: 50 mM HEPES (EuroClone), 1 mM EDTA (Sigma-Aldrich), 10% glycerol (Thermo Fisher Scientific, Waltham, MA, USA), 1% Triton X-100 (Sigma-Aldrich), and 150 mM NaCl in the presence of proteases and phosphatases inhibitors (Sigma-Aldrich). Total protein was measured using the Lowry assay (Bio-Rad DC Protein Assay; Bio-Rad, Hercules, CA, USA) according to manufacturer’s instructions. Protein samples (15 μg) were subsequently loaded onto 4%–20% precast gels (Thermo Fisher Scientific). Gels were electrotransferred onto nitrocellulose membranes (Trans-Blot Turbo Transfer System, Bio-Rad). Membranes were blocked in Tris Buffered Saline (TBS) with 1% Casein-TBS (Bio-Rad) for 1 hour at room temperature and incubated overnight with phospho-ERK (Thr202/Tyr204) (rabbit, 1:1000), phospho-BRAF (Ser445) (rabbit, 1:250), vinculin (mouse, 1:10,000), γ-tubulin (mouse, 1:10,000) (all purchased from Cell Signalling, Leiden, Netherlands), and FLAG tag (mouse, 1:10,000, Sigma-Aldrich) primary antibodies at 4°C. Membranes were washed three times in TBS containing 0.1% Tween and incubated with peroxidase-conjugated secondary antibodies for 45 minutes at room temperature. Bands were visualized using WESTAR Supernova (Cyanagen, Bologna, Italy) and detected with the ChemiDoc™ XRS+ (Bio-rad). After the stripping using RENEW (Cyanagen, Bologna, Italy) buffer, membranes were incubated with total ERK (mouse, 1:1000) and total BRAF (rabbit, 1:250) (Cell Signalling, Leiden, Netherlands) primary antibodies overnight at 4 °C. Membranes were washed three times in TBS containing 0.1% Tween and incubated with peroxidase-conjugated secondary antibodies for 45 minutes at room temperature. Bands were visualized using WESTAR Supernova (Cyanagen, Bologna, Italy) and detected with the ChemiDoc™ XRS+ (Bio-rad). Phosphorylation levels of ERK1/2 and BRAF were evaluated by densitometric analysis of Western Blots performed with ImageLab software (Bio-Rad), and phosphorylated protein was normalized to respective total protein. All data were then normalized to nontransfected controls and reported as the mean ± SEM of at least two independent experiments.

### 4.6. Statistical Analysis

Statistical analysis was carried out using Prism 7 (GraphPad, San Diego, CA, USA). Western Blot experiments were carried out at least in duplicate. Results are expressed as the mean ± SEM. ANOVA test with Tukey’s multiple comparison test was used to analyze differences between groups. A *p*-value < 0.05 (two-tailed) was considered to be statistically significant.

## 5. Conclusions

The analysis of thyroid tissue surrounding PTCs shows how a mutagenic process affecting *BRAF* exon 15 likely takes place in a subset of tumors, leading to the development of p.V600E mutated PTC and to minor histologic alterations of the thyroid parenchyma in the case of non oncogenic BRAF non p.V600E mutations. Our analysis also provides for the first time formal molecular proof that psammoma bodies originate from PTC and represent indeed “the ghosts of dead papillae”.

## Figures and Tables

**Table 1:** Clinicopathologic characteristics of papillary thyroid carcinomas (*n* = 30).

Clinicopathologic Features	Number (%)
Age (years)	47.6 ± 13.6 ^a^
Female sex	20 (66.7%)
Papillary carcinoma	
Classic	24 (80.0%)
Tall cell variant	3 (10.0%)
Follicular variant-infiltrative	1 (3.3%)
Follicular variant-encapsulated with capsular invasion	2 (6.7%)
Tumor size (cm)	1.5 ± 1 ^a^
BRAF p.V600E mutation	20 (66.7%)
Lymph node metastasis	12 (40.0%)
ATA (2015) recurrence risk groups	
Low risk	12 (40.0%)
Intermediate risk	18 (60.0%)
AJCC stage (8th ed.)	
I	29 (96.7%)
II	1 (3.3%)
Other thyroid pathology	
Follicular adenoma	3 (10%)
Follicular adenoma, oncocytic	1 (3.3%)
Chronic thyroiditis	4 (13.3%)
Graves-Basedow disease	1 (3.3%)

^a^ mean ± standard deviation.

**Table 2:** *BRAF* Exon 15 mutations in PTC (*n* = 30) and in adjacent thyroid tissue (*n* = 100).

Samples Analyzed (*n* = 130)	BRAF p.V600E	BRAF non p.V600E	BRAF WT	NA	Total (Samples)
**Papillary carcinoma, all cases (*n* = 30)**	19/130 (14.6%)	1/130 (0.8%)	10/130 (7.7%)	-	30/130 (23.1%)
Papillary carcinoma Classic (*n* = 24)	15/130 (11.5%)	-	9/130 (6.9%)	-	24/130 (18.5%)
Papillary carcinoma Tall cell variant (*n* = 3)	3/130 (2.3%)	-	-	-	3/130 (2.3%)
Papillary carcinoma Follicular variant—infiltrative (*n* = 1)	1/130 (0.8%)	-	-	-	1/130 (0.8%)
Papillary carcinoma Follicular variant—encapsulated with capsular invasion (*n* = 2)	-	1/130 (0.8%) ^a^	1/130 (0.8%)	-	2/130 (1.5%)
**Thyroid tissue adjacent the papillary carcinoma** **(*n* = 100)**					
Psammoma Body (*n* = 6)	3/130 (2.3%)	-	3/130 (2.3%)	-	6/130 (4.6%)
Follicular Adenoma (*n* = 4)	-	1/130 (0.8%)	3/130 (2.3%)	-	4/130 (3.1%)
Oncocytic Follicular Adenoma (*n* = 2)	-	1/130 (0.8%)	1/130 (0.8%)	-	2/130 (%)
Follicular cell atypia (*n* = 38)	-	4/130 (3.1%)	32/130 (24.6%)	2/130 (1.5%)	38/130 (29.2%)
Follicular cell hyperplasia (*n* = 16)	-	2/130 (1.5%)	14/130 (10.8)	-	16/130 (12.3%)
Histologically normal (*n* = 34)	-	1/130 (0.8%)	32/130 (24.6%)	1/130 (0.8%)	34/130 (26.2%)
**Total (Mutation status)**	22/130 (16.9%)	10/130 (7.7%)	95/130 (73.1%)	3/130 (2.3%)	130/130 (100%)

^a^ The single PTC with a BRAF non p.V600E mutation was p.V600A mutated (Case 21, Table S1).

**Table 3:** Type of *BRAF* exon 15 mutations found in thyroid tissue adjacent to PTC and their pathogenicity prediction score (PolyPhen-2).

Thyroid Tissue Samples Adjacent the PTC (*n* = 12)	Mutation	Reference (PMID)	PolyPhen-2 (Score)
**BRAF p.V600E**			
PsB (*n* = 3)	p.V600E	PMID: 15035987	PD (0.971)
**BRAF non p.V600E**			
ATY (*n* = 2), HYP (*n* = 1)	p.G593D	PMID: 19269016	PD (1.000)
ATY (*n* = 1)	p.A598T	PMID: 22926515	PD (0.871)
FA-ONC (*n* = 1)	p.V600K	PMID: 15035987	PD (1.000)
ATY (*n* = 1)	p.V600M	PMID: 28783719	PD (0.904)
FA (*n* = 1)	p.K601E	PMID: 15035987	PoD (0.784)
HYP (*n* = 1)	p.R603Q	PMID: 23861977	PoD (0.786)
NORM (*n* = 1)	p.S607F	PMID: 23861977	PD (0.998)
FA-ONC (*n* = 1), HYP (*n* = 1)	p.S607P	PMID: 29176861	B (0.186)

PsB: psammoma body; FA: follicular adenoma; FA-ONC: follicular adenoma, oncocytic; ATY: thyroid tissue with follicular cell atypia; HYP: thyroid tissue with follicular cell hyperplasia; and NORM: histologically normal thyroid tissue. Two distinct *BRAF* exon 15 mutations were identified in one oncocytic follicular adenoma (p.V600K and p.S607P; Case 4a) and in one focus of follicular cell hyperplasia (p.G593D and p.R603Q; Case 12b). PolyPhen-2 is an in silico tool that predicts mutation pathogenicity assigning the following scores: score 0–0.2, benign (B); score 0.2–0.85, possibly damaging (PoD); and score 0.85–1.00, probably damaging (PD).

**Table 4:** *BRAF* exon 15 mutations in thyroid tissue adjacent to PTC.

***BRAF* Exon 15 Mutations in Thyroid Tissue Adjacent to PTC with the BRAF p.V600E Mutation (*n* = 19)**				
**Total samples (*n* = 71)**	**BRAF p.V600E**	**BRAF non p.V600E**	***BRAF* WT**	**NA**
Psammoma Body (*n* = 3)	3/71 (4.2%)	-	-	-
Follicular Adenoma (*n* = 1)	-	-	1/71 (1.4%)	-
Oncocytic Follicular Adenoma (*n* = 2)	-	1/71 (1.4%)	1/71 (1.4%)	-
Atypical tissue (*n* = 30)	-	4/71 (5.6%)	25/71 (35.2%)	1/71 (1.4%)
Hyperplasia (*n* = 12)	-	2/71 (2.8%)	10/71 (14.1%)	-
Normal tissue (*n* = 23)	-	1/71 (1.4%)	21/71 (29.6%)	1/71 (1.4%)
***BRAF* Exon 15 Mutations in Thyroid Tissue Adjacent to PTC *BRAF* Exon 15 Wild Type (*n* = 10) or with BRAF non p.V600E Mutation (*n* = 1)**				
**Total samples (*n* = 29)**	**BRAF p.V600E**	**BRAF non p.V600E**	***BRAF* WT**	**NA**
Psammoma Body (*n* = 3)	-	-	3/29 (10.3%)	-
Follicular Adenoma (*n* = 3)	-	1/29 (3.5%)	2/29 (6.9%)	-
Atypical tissue (*n* = 8)	-	-	7/29 (24.1%)	1/29 (3.5%)
Hyperplasia (*n* = 4)	-	-	4/29 (13.8%)	-
Normal tissue (*n* = 11)	-	-	11/29 (37.9%)	-

**Table 5:** Primers used for sequencing and mutagenesis.

***BRAF* (Exon 15) Primers for Next Generation Sequencing**		
*BRAF* (Exon 15)	FW:	TGCTTGCTCTGATAGGAAAATGA
	RV:	TGGATCCAGACAACTGTTCAAA
***BRAF* (Exon 15) Primers for Mutagenesis**		
p.G593D	FW:	GTAAAAATAGATGATTTTGGTCTAGC
	RV:	TGTGAGGTCTTCATGAAG
p.A598T	FW:	TTTTGGTCTAACTACAGTGAAATC
	RV:	TCACCTATTTTTACTGTGAG
p.V600E	FW:	CTAGCTACAGAGAAATCTCGATG
	RV:	ACCAAAATCACCTATTTTTAC
p.K601E	FW:	AGCTACAGTGGAATCTCGATG
	RV:	AGACCAAAATCACCTATTTTTAC
p.S607F	FW:	TGGAGTGGGTTCCATCAGTTT
	RV:	TCGAGATTTCACTGTAGCTAG
p.S607P	FW:	ATGGAGTGGGCCCCATCAGTT
	RV:	CGAGATTTCACTGTAGCTAG
***BRAF* (Exon 15) Primers for Sanger Sequencing**		
*BRAF* (Exon 15)	FW:	ACACGCCAAGTCAATCATCC
	RV:	TCTGACTGAAAGCTGTATGGATT

In bold: nucleotide changes for mutagenesis.

## Supplementary Materials

The following are available online at https://www.mdpi.com/2072-6694/12/2/430/s1. Figure S1: Western Blot analysis of ERK and BRAF, Table S1: Next generation sequencing results of individual samples, Table S2: Western Blot densitometry reading ratios of ERK and BRAF.
